# Making BREAD: Biomimetic Strategies for Artificial Intelligence Now and in the Future

**DOI:** 10.3389/fnins.2019.00666

**Published:** 2019-06-27

**Authors:** Jeffrey L. Krichmar, William Severa, Muhammad S. Khan, James L. Olds

**Affiliations:** ^1^Departments of Cognitive Sciences and Computer Science, University of California, Irvine, Irvine, CA, United States; ^2^Sandia National Laboratories, Data-Driven and Neural Computing, Albuquerque, NM, United States; ^3^Schar School, George Mason University, Arlington, VA, United States

**Keywords:** AI, biomimetic, energy, edge computing, neurobiology, neuromorphic computing

## Abstract

The Artificial Intelligence (AI) revolution foretold of during the 1960s is well underway in the second decade of the twenty first century. Its period of phenomenal growth likely lies ahead. AI-operated machines and technologies will extend the reach of Homo sapiens far beyond the biological constraints imposed by evolution: outwards further into deep space, as well as inwards into the nano-world of DNA sequences and relevant medical applications. And yet, we believe, there are crucial lessons that biology can offer that will enable a prosperous future for AI. For machines in general, and for AI's especially, operating over extended periods or in extreme environments will require energy usage orders of magnitudes more efficient than exists today. In many operational environments, energy sources will be constrained. The AI's design and function may be dependent upon the type of energy source, as well as its availability and accessibility. Any plans for AI devices operating in a challenging environment must begin with the question of how they are powered, where fuel is located, how energy is stored and made available to the machine, and how long the machine can operate on specific energy units. While one of the key advantages of AI use is to reduce the dimensionality of a complex problem, the fact remains that some energy is required for functionality. Hence, the materials and technologies that provide the needed energy represent a critical challenge toward future use scenarios of AI and should be integrated into their design. Here we look to the brain and other aspects of biology as inspiration for Biomimetic Research for Energy-efficient AI Designs (BREAD).

## Artificial Intelligence's Energy Requirements

The last few years have seen a rapid expansion of Artificial Intelligence (AI) and Machine Learning (ML) breakthroughs. What were once AI solutions to toy problems have now become human level complex problem-solving. These solutions have moved out of research labs and into commercial applications. However, most AI and ML algorithms for these complex problems are implemented in large data centers housing power hungry clusters of computers and Graphical Processing Units (GPUs). In contrast, natural, biological intelligence is power efficient and self-sufficient. In this article, we argue for Biomimetic Research for Energy-efficient AI Designs (BREAD) as AI moves toward edge computing in remote environments far away from conventional energy sources, and as energy consumption becomes increasingly expensive.

###  Current Solutions to AI's Energy Requirements

**Figure 1:**
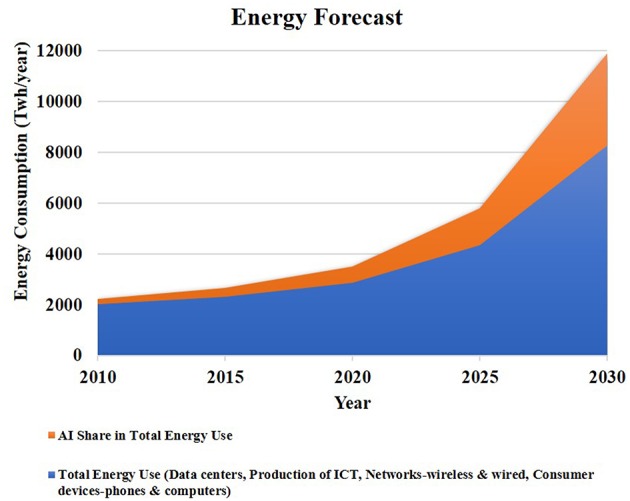
The graph shows that total energy use and share of AI in the total energy use will increase in the 2020s. The AI share in total energy use is on the order of 40% by 2030. Trends estimated from Andrae and Edler (2015) and IEA (2017).

With the growth of the Internet, data traffic (traffic to and from data centers) is escalating exponentially, crossing a zettabyte (1.1 ZB) in 2017 (Andrae and Edler, 2015). Figure 1 shows this trend. Currently, data centers consume an assessed 200 terawatt hours (TWh) each year equivalent to 1% of global electricity demand (Jones, 2018). A 2017 International Energy Agency (IEA) report noted that with the ongoing explosion of Internet traffic in data centers, electricity demand will likely to increase by 3% (IEA, 2017). While it is difficult to estimate the exact role of AI within data centers, analysis from reports (Andrae and Edler, 2015; Sverdlik, 2016; IEA, 2017; Gagan, 2018; CBInsights, 2019) suggests that it is non-trivial— on the order of 40% (see Figure 1). For example, Google projected in 2013 that people searching by voice for three minutes a day using speech recognition deep neural networks would double their datacenters' computation demands, and this was one impetus for developing the Google TPU (Jouppi et al., 2017). Additionally, Facebook has stated that machine learning is “applied pervasively across nearly all sevices” and that “computational requirements are also intense” (Hazelwood et al., 2018).

The rise of highly efficient data factories, known as hyperscale facilities, use an organized uniform computing architecture that scales up to hundreds of thousands of servers. While these hyperscale centers can be optimized for high computing efficiency, there are limits to growth due to a variety of constraints that also affect other electrical grid consumers. However, the shift to hyperscale facilities is a current trend, and if 80% of servers in US conventional data centers were moved over to hyperscale facilities, energy consumption would drop by 25%, according to Lawrence Berkeley National Laboratory report, 2016.

One way the hyperscale centers have cut down their power usage is through efficiencies in cooling. By locating in cooler climates, the data centers can ingest the cool air outside with positive results. Another solution is employing warm water cooling loops, a solution tuned for temperate and warm climates. An innovative solution to address the energy constraints of AI systems is to employ an AI-powered cloud-based control recommendation system. For example, Google employs a cloud-based AI to collect information about the data center cooling system from thousands of physical sensors prior to feeding this information into deep neural networks. The networks then compute predictions for how different combinations of possible activities will affect future energy consumption (Shehabi et al., 2016).

Although hyperscale centers and smart cooling strategies can lower energy consumption, these solutions do not address applications where AI is operating at the edge or when AI is deployed in extreme conditions far away from convenient power supplies. We believe that this is where future AI systems are headed.

Our view is that there is a pressing need to address the energy issue as it applies to the future of AI and ML. While there is a growing research effort toward developing efficient machine learning methods for embedded and neuromorphic processors (Esser et al., 2015; Hunsberger and Eliasmith, 2016; Rastegari et al., 2016; Howard et al., 2017; Yang et al., 2017; Severa et al., 2019), we recognize that these methods do not address the full needs of future applications, despite offering compelling first steps. Generally, current methods modify existing techniques rather than develop de novo algorithms.

In this paper, we emphasize how biology has addressed the power consumption problem, with a particular focus on energy efficiency in the brain. Furthermore, we look at non-neural aspects of biology that also lead to power savings. We suggest that these strategies from biology can be realized in future AI systems.

## Current State of AI as it Pertains to Energy Consumption

###  Edge Computing in Remote and Hostile Environments

Trends in many human-built systems point to directions where sensing, processing, and actuation is situated on distributed platforms. The emerging Internet-of-Things (IoT) are cyber technologies (Atzori et al., 2010), hardware and software, that interact with physical components in environments which may or may not be populated by humans. IoT devices are often thought of as the “edge” of a large, sophisticated cloud processing infrastructure. Processing data at the “edge,” reduces system latency by removing the delays in the aggregation tiers of the information technology infrastructure (Hu et al., 2015; Mao et al., 2016; Shi et al., 2016). In addition to minimizing latency, edge processing increases system security and mitigates privacy concerns when processing data in the cloud. In cases where the data path between the edge and user is very long, such computation can, by feature extraction, reduce the dimensionality and hence the expense of sending information. However, edge processing may be far away from power sources and may need to operate without intervention over long time periods.

Exploration of remote and hostile environments, such as space and deep ocean, will most likely require AI and ML solutions. These environments are inherently hostile to the circuitry that sub-serves current AI and ML technologies.

Unless human beings can be “radiation-hardened,” robotic space probes will continue to dominate exploration and exploitation of space in domains ranging from low earth orbit to interstellar exploration. All of these are subject to a variety of hazards which are potentially hazardous to CMOS-based AI. These include collisions with high energy photons (such as gamma rays), micrometeorites, planetary weather, and anthropogenic attacks. Planetary missions such as NASA's Curiosity Mars rover have revealed additional challenges from weather (such as sandstorms) that have put missions at risk. Radioisotope thermal generation (RTG) power was added to the Curiosity Mars rover design to combat the vulnerabilities to solar energy systems on previous missions. While Curiosity's computational systems do not constitute true AI, the power demands of the entire Curiosity rover (including drills and actuators) are of a similar order of magnitude. Recent concerns over limitations on the availability of radioisotopes (Aebersold, 1949) combined with safety concerns (Staff, 1948; Al Kattar et al., 2015) during the launch phase will constrain future deep space missions that might use nuclear power (Billings, 2015; Grush, 2018; Lakdawalla, 2018; Grossman, 2019).

As with Space, in deep ocean environments, power constraints are also a current challenge. Current non-nuclear powered Autonomous Underwater Vehicles (AUVs) have limited capabilities due to restrictions on energy storage and the availability of fuel sources. Furthermore, the extremely high pressures of deep-sea environments offer their own challenges, not only to energy supply for AI but also to the mass and construction of protection containers for the electronics. Ocean glider AUV's use buoyancy engines with fins to convert force in the vertical direction to horizontal motion (Webb et al., 2001; Schofield et al., 2007; Rudnick, 2016; Rudnick et al., 2016, 2018). While very slow, such AUV's are far less energy-constrained than other current technologies. However, the power generated by such engines is not currently suitable for powering AI systems. Batteries are used for such functions and must be recharged at the ocean surface using photo-voltaic cells. There are proposals to use nuclear fission power generation to enable deep-sea battery recharging stations for military AUV's, though these remain at the development stage and have similar safety considerations to those mentioned above for space (Hambling, 2017).

In many of these domains, AI will be the preferred computational modality because of latency issues related to long-distance communication with Earth-based controllers. Operating in such domains will have the additional challenge of energetic constraints because readily available solar power may not always be available in domains such as Earth's moon, solar system planets with weather and deep space (including interstellar). The primary alternative energy source for such domains is nuclear (both fission and fusion-based). Such power sources are in contrast to the current radioisotope thermo-electric technologies used for missions such as the Mars Curiosity Rover. While break-even fusion power has yet to be demonstrated on Earth, the abundance of fusion fuels in the solar system makes such power sources attractive. In all these cases, the nuclear technology must have a similar resiliency to that of the AI in terms of hazards, and it will be optimal to consider such requirements holistically at the design stage.

###  Existence Proof, Human Brains as Efficient Energy Consumers

The original goal of AI was to extract principles from human intelligence. On the one hand, these principles would allow for a better understanding of intelligence and what makes us human. On the other hand, we could use those principles to build *intelligent* artifacts, such as robots, computers, and machines. In both cases, the goal is to use human intelligence as a use case, which derives from the function of the brain. We believe that there are also important energy efficiency principles that can be extracted from neurobiology and applied to AI. Therefore, the nervous system can provide much inspiration for the construction of low power intelligent systems.

The human nervous system is under tight metabolic constraints. These constraints include the essential role of glucose as fuel under conditions of non-starvation, the continuous demand for approximately 20% of the human body's total energy utilization, and the lack of any effective energy-reserve among others (Sokoloff, 1960). And yet, as is well known, the brain operates on a mere 20 W of power, approximately the same power required for a ceiling fan operating at low speed. While being severely metabolically constrained is at one level a disadvantage, evolution has optimized brains in ways that lead to incredibly efficient representations of important environmental features that stand distinct from those employed in current digital computers.

The human brain utilizes many means to reduce functional metabolic energy utilization. Indeed, one can observe at every level of the nervous system strategies to maintain high performance and information transfer, while minimizing energy expenditure. These range from ion channel distributions, to coding methods, to wiring diagrams (connectomes). Many of these strategies could inspire new methodologies for constructing power efficient artificial intelligent systems.

At the neuronal coding level, the brain uses several strategies to reduce neural activity without sacrificing performance. Neural activity, (i.e., the generation of an action potential, the return to resting state, and synaptic processing) is energetically very costly, and this can drive the minimization of the number of spikes necessary to encode either an engram or the neural representation of a new stimulus (Levy and Baxter, 1996; Lennie, 2003). Such sparse coding strategies appear to be ubiquitous throughout the brain (Olshausen and Field, 1997, 2004; Beyeler et al., 2017).

Furthermore, dimensionality reduction methods from machine learning can explain many neural representations (Beyeler et al., 2016). Because brains face strict constraints on metabolic cost (Lennie, 2003) and anatomical bottlenecks (Ganguli and Sompolinsky, 2012), which often force the information stored in a large number of neurons to be compressed into an order of magnitude smaller population of downstream neurons (e.g., storing information from 100 million photoreceptors in 1 million optic nerve fibers), reducing the number of variables required to represent a particular stimulus space figures prominently in efficient current coding theories of brain function (Linsker, 1990; Barlow, 2001; Atick, 2011). Such views posit that the brain performs dimensionality reduction by maximizing mutual information between the high-dimensional input and the low-dimensional output of neuronal populations. Although dimensionality reduction is typically used in machine learning to improve generalization, it may have implications for energy efficiency in real and artificial neural networks. By decreasing the number of neurons required to represent stimuli, while adhering to sparsity constraints, energy savings can be achieved without loss of information.

The brain must respond quickly to stimuli and changes in the environment. However, this implies an increase in neural activity, which would be energetically costly. Evidence suggests that this is not the case and that the brain utilizes strategies to maintain a constant rate of activity. For example, the nervous system can respond quickly to perturbations by shifting the specific timing rather than increasing the absolute number of spikes (Malyshev et al., 2013). Moreover, the balance of excitation and inhibition can further maintain a steady rate of neural activity while still being responsive (Sengupta et al., 2013a; Yu et al., 2018). In these ways, the overall energy utilization of the human brain stays relatively constant, while the local rate of energy consumption varies widely and is dependent upon functional neuronal activity and the balance between excitatory and inhibitory neurons (Olds et al., 1994). In a similar way, neural networks and neuromorphic hardware may reduce the activity of unused or unnecessary nodes, when other nodes are highly active. Thus, keeping the overall power budget constant. For example, the SpiNNaker system will turn off turn off cores when they are not needed (Furber et al., 2013). This strategy may be applied dynamically during operation.

At a macroscopic scale, the brain saves energy by minimizing the wiring between neurons and brain regions (i.e., number of axons), yet still communicates information at a high-level of performance (Laughlin and Sejnowski, 2003). Unlike current electronic chips, the brain packs its wiring into a three-dimensional space, which not only reduces the overall volume but also can reduce the energy cost. Energy is further conserved by maintaining high local connectivity with sparse distal connectivity. White matter, which are myelinated axons that transmit information over long distances in the nervous system, make up about half the human brain but use less energy than gray matter (neuronal somata and dendrites) because of the scarcity of ion channels along these axons (Harris and Attwell, 2012). These myelinated axons speed up signal propagation and reduce the volume of matter in the brain. However, information transfer between neurons and brain areas is still preserved by the overall architecture, which essentially is a small world network (Sporns and Zwi, 2004; Sporns, 2006). That is, even though the probability of any two distal cortical neurons being connected is extremely low, any two neurons are only a few connections away from each other.

The nervous system also optimizes energy consumption at the cellular and sub-cellular levels. Minimizing wiring has energy implications for both hardware and software. In hardware, routing of information within and between processors can have an impact on energy consumption. In software, the handling of synapses, as is in the brain, takes the most processing power. There are typically many more synaptic events to handle than neuron updates. Minimizing the wiring or number of connections could potential yield energy savings.

It has been suggested that the brain strives to minimize its free energy by reducing surprise and predicting outcomes (Friston, 2010). Thus, the brain's efficient power consumption may have a basis in thermodynamics and information theory. That is, the system may adapt to resist a natural tendency toward disorder in an ever-changing environment. Top-down signals from downstream areas (e.g., frontal cortex or parietal cortex) can realize predictive coding (Clark, 2013; Sengupta et al., 2013a,b). In this way organisms minimize the long-term average of surprise, which is the inverse of entropy, by predicting future outcomes. In essence, they minimize the expenditures required to deal with unanticipated events. The idea of minimizing free energy has close ties to many existing brain theories, such as the Bayesian brain, predictive coding, cell assemblies, and Infomax, as well as an evolutionary-inspired theory called Neural Darwinism or neuronal group selection (Friston, 2010). For field robotics, a predictive controller could allow the robot to reduce unplanned actions (e.g., obstacle avoidance) and produce more efficient behaviors (e.g., optimal foraging or route planning). For IoT and edge processing, predictions could reduce communication data. Rather than sending redundant predictable information, it would only need to “wake up” and report when something unexpected occurs.

In summary, the brain represents an important existence proof that extraordinarily efficient natural intelligence can compute in very complex ways within harsh, dynamic environments. Beyond an existence proof, brains provide an opportunity for reverse-engineering in the context of machine learning methods and neuromorphic computing.

###  Energy Efficiency Through Brain-Inspired Computing

A key component in pursuing brain- and neural- inspired computing, coding, and neuromorphic algorithms lies in the currently shifting landscape of computing architectures. Moore's law, which has dictated the development of ever-smaller transistors since the 1960s, has become more and more difficult to follow, leading many to claim its demise (Waldrop, 2016). This has inspired renewed interest in heterogeneous and non-Von Neumann computing platforms (Chung et al., 2010; Shalf and Leland, 2015), which take inspiration from the efficiency of the brain's architecture. Neuromorphic architectures can offer orders-of-magnitude improvement in performance-per-Watt compared to traditional CPUs and GPUs (Indiveri et al., 2011; Hasler and Marr, 2013; Merolla et al., 2014). However, the benefit of neuromorphic computing can and will depend on the application chosen. For modeling biological neural systems, the performance improvements already may be considerable (e.g., the Neurogrid platform claims 5 orders of magnitude efficiency improvement compared to a personal computer Benjamin et al., 2014). Additionally neural approaches enable IBM's TrueNorth chip to power convolutional neural networks for embedded gesture recognition at less than one Watt (Amir et al., 2017). In another comparison, a collection of image (32 × 32 pixel) benchmark tasks on TrueNorth resulted in approximately 6,000 to 10,000 frames/Second/Watt, whereas the Nvidia Jetson TX1 (an embedded GPU platform) can process between 5 and 200 (ImageNet) frames per second at approximately 10–14 Watts net power consumption (Canziani et al., 2016). Although we note that it is difficult to have fair network and dataset parity across platforms, and that neuromorphic systems supporting even millions of neurons may be too small-scale for application-level machine learning problems.

**Figure 2:**
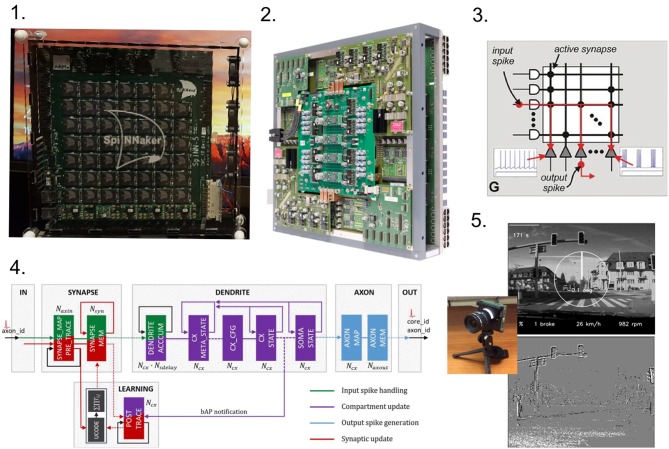
Various neuromorphic platforms are pictured. **(1)** SpiNNaker 48-node board utilizes ARM chips to calculate neuron dynamics (Furber et al., 2013). **(2)** A fully assembled BrainScaleS wafer module. Image from Schmitt et al. (2017). **(3)** Schematic of the functional crossbar representation of a IBM TrueNorth core. Image from Merolla et al. (2014). **(4)** Neuromorphic core structure on Intel Loihi consists of four main computing modes, from Davies et al. (2018). **(5)** Frames (annotated with driving data; top) and events (bottom) recoded on a retina-inspired DAVIS sensor (Brandli et al., 2014), similar to that pictured in the inset. Sample images from Binas et al. (2017).

Neuromorphic architectures refer to a wide variety of computing hardware platforms (Schuman et al., 2017), from sensing (Liu and Delbruck, 2010; Posch et al., 2014) to processing (Indiveri et al., 2011; Merolla et al., 2014), analog (Fieres et al., 2008) to digital (Furber et al., 2013; Merolla et al., 2014); see Figure 2. However, in most cases the defining characteristics take inspiration from the brain:

1. Massively parallel, simple integrating processing units (neurons)
2. Sparse and dynamic low-precision communication via “spikes.”
3. Event-driven, asynchronous operation.

This event-driven, distributed, processor-in-memory approach provides robust, low-power processing compatible with many neural-inspired machine learning and artificial intelligence applications (Neftci, 2018). Hence, size, weight and power (SWaP) constrained environments, such as edge and IoT devices, can leverage increased effective remote computation capabilities and provide real-time, low-latency intelligent and adaptive behavior. Moreover, the often noisy nature of learned artificial intelligence systems (some incorporate noise by design Srivastava et al., 2014) may lead to more robust computation in extreme environments such as space.

Heterogeneous (spiking and non-spiking) architectures are improving performance and latency, exemplified by a 30–80x improvement on deep learning tasks (Putnam et al., 2014; Jouppi et al., 2017), and new neuromorphic architectures, such as Intel's Loihi, are themselves heterogeneous which improves communication between neural and conventional cores (Davies et al., 2018). Emerging neural computing platforms may benefit traditional large-scale computation both indirectly [e.g., system health (Das et al., 2018), failure prediction (Bouguerra et al., 2013)] and directly (e.g., meshing, surrogate models Melo et al., 2014), and recent work indicates that neuromorphic processors may be useful for direct computation due their high-communication, highly-parallel nature (Lagorce and Benosman, 2015; Jonke et al., 2016; Aimone et al., 2017; Severa et al., 2018). However, we do remark that currently several challenges exist hindering wide-range adoption of these platforms. Some of the primary difficulties include: (1) Neuromorphic chips are a niche product and difficult to procure at volume; (2) There is insufficient software interfaces for developing applications; (3) Many algorithms are incompatible or may underperform on neuromorphic hardware; (4) Large-scale applications are often too large; (5) Cross-compiling code and I/O require considerable time and bandwidth from a host machine. See Diamond et al. (2016), Severa et al. (2019), Hunsberger and Eliasmith (2016), Disney et al. (2016), Davison et al. (2009), Ehsan et al. (2017), and Wolfe et al. (2018) for more details and possible approaches toward solving these challenges. Moreover, some of the themes from the *Existence proof, human brains as efficient energy consumers* section (e.g., minimizing wiring, keeping firing rates constant, using sparse and reduced representations) could be incorporated into neuromorphic designs.

Neural inspiration has also impacted data collection in the form of spiking neuromorphic sensors which generally follow the same three characteristics as neuromorphic architectures. The two most common categories are silicon cochleas (Watts et al., 1992; Chan et al., 2007) and retina-inspired event-driven cameras (Delbrück et al., 2010; Delbruck et al., 2014), though neuromorphic olfaction is also under active research and development (Vanarse et al., 2016). Neuromorphic sensors can often be thought of as a method for high-speed preprocessing, fundamentally changing the sample space. For example, for imagery this allows for low-bandwidth, high-sampling, and high-dynamic range imagery (Delbrück et al., 2010; Posch et al., 2015). These benefits, in turn, have enabled low-latency, low-power applications such as gesture recognition (Ahn et al., 2011; Amir et al., 2017), robotic control (Conradt et al., 2009; Delbruck and Lang, 2013), and movement determination (Drazen et al., 2011; Haessig et al., 2018). The sparse, spiking representations can pose an algorithmic challenge, at times being incompatible with common processing methods designed around rasterized data. However, spiking sensors are innately compatible with spiking neuromorphic processors, and combining neuromorphic sensors with a neuromorphic processor can avoid the costly conversion between binary data formats and spikes.

Computational requirements of artificial intelligence algorithms limit their remote applications today. Consequently, most current consumer or commercial machine learning technologies are reliant on connections to remote data centers. However, as neuromorphic technologies transition from research platforms to everyday products, learning systems can and will proliferate in capability and scope. Combined with the expected growth of edge and IoT devices, we can expect persistence and pervasive learning devices. These learning devices, extensions of current trends in smart devices (e.g., digital assistants, smart home control, wearables), will be enhanced with personalized online learning and enabled with adaptive, intelligent and context-dependent perception and behaviors. Ultra-low energy neuromorphic chips will carry out computations using milliwatts of power. In the industrial, medical and security spaces, the same technologies will provide low-powered sensors capable of extended deployment in a variety of extreme environments.

**Figure 3:**
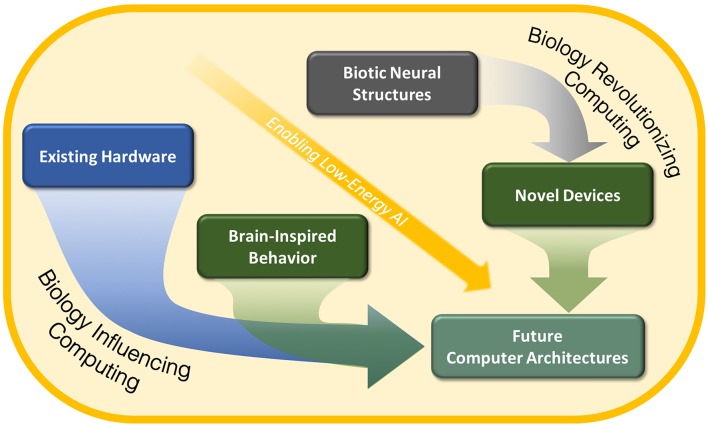
The image shows that in the design of bio-mimetic circuitry, either the existing hardware will be slightly adjusted to copy the behavior of brain parts, or new computing architecture will be designed so that it completely emulate the high energy-efficient biotic neural structures (Image adapted from Calimera et al., 2013).

There is ample precedent for brain-inspired approaches to engineering design that may lead to energy-efficient AI and edge computing. Both designing algorithms to mimic the brain's behavior, and building new computer hardware that mimic neural dynamics can lead to energy efficiency (see Figure 3) (Calimera et al., 2013). However, many of the brain's energy efficiency strategies, such as minimizing wiring, maintaining constant activity and prediction outcomes, are not implemented in current neuromorphic architectures and should be explored in the future.

## Other Energy Efficient Strategies in Biology

Energy efficiency can also be inspired by observing nature's non-neural solutions. For example, the wing of an aircraft takes inspiration from the wings of flying animals (birds, bats, and insects). The shape of a modern naval submarine has evolved from early boat-like designs prevalent during the First and Second World Wars toward a more streamlined whale-like shape. Even DNA-based computation–by itself incredibly energetically efficient–takes inspiration from the conserved phylogenetic information transfer mechanism of Earth's biosphere. The adaptive immune system, with its sophisticated “learning and memory” through selection also represents a low-energy approach to artificial intelligence that may eventually have applications to AI-enhanced cyber-security applications (Forrest, 1993; Somayaji et al., 1998; Forbes, 2004; Forrest and Beauchemin, 2007; Rice and Martin, 2007; Keller, 2017). This selectionist approach, which was inspired by the immune system, led to an influential brain theory where the synaptic selection took place during neural development and through experiential synaptic plasticity (Edelman, 1987, 1993). Such a Darwinist approach can lead to efficient neural network structures.

**Figure 4:**
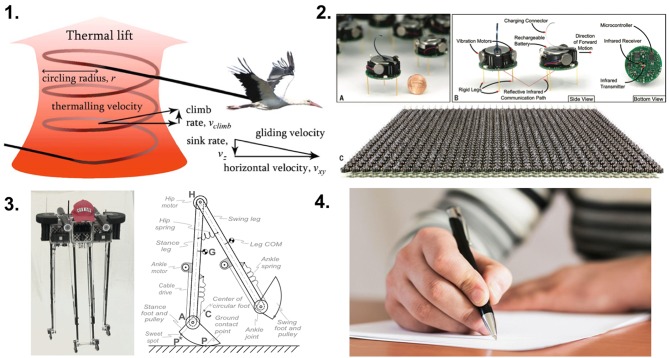
Examples of morphological computation in nature and in engineering. **(1)** Thermal soaring is a form of flight where birds can stay in the air without providing power from flapping. From Akos et al. (2010). **(2)** A robotic swarm inspired by social insects. From Rubenstein et al. (2014). **(3)** An efficient walking robot that exploits passive dynamics. From Bhounsule et al. (2014). **(4)** The dexterity of the human hand is realized with soft skin and high resolution touch receptors at the fingertips. From Balter (2015).

As edge computing and mobile sensing devices become ubiquitous, efficient mobility, whether on land, air, or water will become increasingly important. Figure 4 shows examples of how biological organisms have evolved to leverage their environment, and this morphological computation can lead to efficient movement and information processing (Pfeifer et al., 2014). For example, swarm intelligence (see Figure 4, panel 2), which is inspired by social insects, can solve a number of problems with a collection of low power simple agents. Interesting solutions emerge through the agents' interactions Rubenstein et al. (2014).

Bipedal walking is somewhat of a controlled fall, where energy is conserved by allowing gravity to take over after the swing phase of a step (see Figure 4, panel 3). This strategy has been adopted in passive walker robots that utilize orders of magnitude less energy than conventional walking robots (Collins et al., 2005; Bhounsule et al., 2014). Birds of prey and long-range migrating birds take advantage of thermal plumes to reduce energy usage during flight (see Figure 4, panel 1) (Akos et al., 2010; Weimerskirch et al., 2016; Bousquet et al., 2017). Gliders have mimicked this strategy in their flight control systems (Allen and Lin, 2007; Edwards, 2008; Reddy et al., 2018). Similar to many fish and marine mammals, oceanographic submersible gliders can harvest energy from the heat flow of thermal gradients (Webb et al., 2001; Schofield et al., 2007; Rudnick, 2016; Rudnick et al., 2016, 2018). These submersible gliders can operate across thousands of kilometers over months to years. Some fish species and flying insects alter their environment (i.e., the water or air vortices) to create additional thrust (Triantafyllou et al., 2002; Sane, 2003). The human hand is a marvel of morphological computing. The shape of the hand naturally and reflexively grasps onto object. The first point of contact is high resolution sensors made of compliant material (i.e., the fingertips). Such structures greatly reduce the neural computing load for complex tasks (see Figure 4, panel 4).

Inspiration from biology at the population scale can lead to efficient solutions to problems. Social insects and bacterial colonies have inspired highly distributed robots or computing systems (Rubenstein et al., 2014; Werfel et al., 2014). In these cases, each agent has very low power computation requirements, and no single agent is a point of failure. However, the interactions between these agents can lead to complex problem-solving, which is sometimes referred to as swarm intelligence.

Taken together, future AI systems that take inspiration from biology and other energy harvesting approaches will have a distinct advantage for long-term operation in harsh or remote environments. Following these biological strategies could allow for energy efficient sensor networks at the edge, more efficient manufacturing, and systems that operate over much longer timescales.

## Conclusions

AI is on a trajectory to fundamentally change society in much the same way that the industrial revolution did. Even without the development of General Artificial Intelligence, the trend is toward human-machine partnerships that collectively will have the ability to substantially extend the reach of humans in multiple domains (e.g., space, cyber, deep sea, nano). However, as with many things, there is no free lunch: AI will require energy inputs that we believe must be accounted for at all stages of the AI design process. We believe that such design solutions should leverage the solutions that biology, especially the human brain, has evolved to be energy efficient without sacrificing functionality. These solutions are critical components to what we call *intelligence*.

AI has the potential to change society drastically; the evolving human-machine partnerships will substantially extend the reach of humans in multiple domains. For this to happen, AI will require energy inputs that must be an early component of future integrated AI design processes. A coherent strategy for design solutions should leverage the solutions that biology, especially biological brains, offers in maintaining energy efficiency and preserving functionality. This strategy advances the following recommendations to ensure both private and government support for research and innovation.

###  Recommendation 1: A Multinational Initiative to Make BREAD

To coordinate investments and channel knowledge from the life sciences to AI energetics into a holistic AI design, we advocate the launch of a global technological innovation initiative, which we call Biomimetic Research for Energy-efficient, AI Designs (BREAD). Ideally, BREAD would be backed by professional societies such as the Association for Computing Machinery (ACM), American Psychological Association (APA), Institute of Electrical and Electronics Engineers (IEEE), and Society for Neuroscience, as well as federal agencies. Energy sustainability would be central in BREAD, but the initiative would encompass all aspects of AI from hardware to sensors.

###  Recommendation 2: Integrate Biomimetic Energetic Solutions Into Future AI Designs

Future AI development will require an integrated design process where energy supply is not an add-on or assumed. Thermodynamic considerations alone make the energetic considerations important, particularly for those at the Edge, such as IoT and Space environments, which are inherently hostile to CMOS or future successor chip technologies. Current AI, such as deep learning, approaches the problem by situating data centers close to abundant and cheap electric power sources much like what Google, Facebook, and IBM do. We believe that a more fruitful approach for AI design is to leverage the solutions evolved by biology (nervous system, metabolism, morphology) in the future AI design, to what we call ‘biomimetic strategies.'

###  Recommendation 3: An Industry Backed Research Lab or Consortium

Since the initial costs of integrating biomimetic solutions into AI are likely to be front-loaded, we recommend that stakeholder industrial partners with governments establish a pre-competitive research laboratory that preserves intellectual property, similar to IMEC in Belgium. IMEC was created so that CMOS design firms might prototype new chips in a state-of-the-art environment. Alternatively, an industry backed research and development of technology, similar to the Semiconductor Research Consortium (SRC), would be another model to move forward on energy efficient bio-inspired AI solutions. Such models could catalyze the technological innovations necessary for success.

###  Recommendation 4: A Trainee Pipeline

A critical component of BREAD would be to establish a pool of scientists at the intersection of AI and energy issues who can integrate knowledge from biology, computer science, neuroscience, and engineering. Thus, aligned with BREAD, research institutions should consider new graduate offerings at this nexus. In the US, the Engineering Directorate of the NSF, the DOE or the DOD might support doctoral candidates and post-doctoral trainees in this area through fellowships and scholarships.

In conclusion, we see the future development of AI as requiring new strategies for embedding energy demands of the machine into the overall design strategy. From our standpoint, this must include biomimetic solutions. As indicated above, there is much precedence for this type of engineering in other high aspects of technology, especially those that must operate in challenging environments. Now such engineering must be applied to future AI design so that the technological trajectory of this paradigm-changing technology is secure.

## Author Contributions

JK, WS, MK, and JO wrote the manuscript.

### Conflict of Interest Statement

The authors declare that the research was conducted in the absence of any commercial or financial relationships that could be construed as a potential conflict of interest.
